# Optimising the laboratory supply chain: The key to effective laboratory services

**DOI:** 10.4102/ajlm.v3i1.101

**Published:** 2014-09-05

**Authors:** Dianna Edgil, Jason Williams, Peter Smith, Joel Kuritsky

**Affiliations:** 1 United States Agency for International Development (USAID), Washington, United States; 2 The Partnership for Supply Chain Management (PFSCM), Arlington, United States

## Abstract

**Background:**

The Supply Chain Management System (SCMS) is a contract managed under the Partnership for Supply Chain Management (PFSCM) consortium by the United States Agency for International Development (USAID). SCMS procures commodities for programmes supported by the US President’s Emergency Plan for AIDS Relief (PEPFAR). From 2005 to mid-2012, PEPFAR, through SCMS, spent approximately $384 million on non-pharmaceutical commodities. Of this, an estimated $90m was used to purchase flow cytometry technology, largely for flow cytometry platforms and reagents.

**Objectives:**

The purpose of this paper is to highlight the cost differences between low, medium and high utilisation rates of common CD4 testing instruments that have been procured though PEPFAR funding.

**Method:**

A scale of costs per test as a function of test volume through the machine was calculated for the two most common CD4 testing machines used in HIV programmes: Becton Dickinson (BD) FACSCount™ and BD FACSCalibur™. Instrument utilisation data collected at the facility level in three selected countries were then used to calculate the onsite cost-per-test experienced in each country.

**Results:**

Cost analyses indicated that a target of at least 40% utilisation for FACSCount™ and 15% utilisation for FACSCalibur™, respectively, closely approach maximal per-test cost efficiency. The average utilisation rate for CD4 testing instruments varies widely by country, level of laboratory and partner (0% − 68%).

**Conclusion:**

Our analysis indicates that, because cost-per-test is related inversely to sample throughput, the underutilisation of flow cytometry machines is resulting in an increase in average cost-per-test for many instruments.

## Introduction

The US President’s Emergency Plan for AIDS Relief (PEPFAR) has directed significant resources toward diagnosing and treating HIV in selected developing countries. In support of this programme, the United States government established a supply chain contract mechanism that is implemented by the Partnership for Supply Chain Management (PFSCM) and managed by the United States Agency for International Development (USAID). The purpose of this contract is to provide technical assistance to host countries with procurement of pharmaceuticals, laboratory products and other items for country programmes.

The global HIV community has recognised that increasing the efficiency of current programmes through better management, data-driven decision making and appropriate resource allocation are key to the continued scale-up of HIV programmes necessary for universal access to care and treatment.^1,2^

Laboratory diagnostic services are a critical component of HIV programmes and are key to programmes’ ability to scale up treatment for HIV.^3^ Building laboratory capacity requires resources and is accompanied by supply chain challenges.^4,5^ Optimising laboratory procurement by using an evidence-based strategy to inform the procurement of laboratory instruments that are appropriate to the throughput (i.e., tier) of the laboratory in which they are to be placed, offers an opportunity to increase value for money^6^ and ensures that programmes can maximise the number of patients with access to reliable laboratory diagnostic services. The purpose of this article is to analyse costs associated with flow cytometry platforms by studying utilisation rates in three countries and comparing them to the maximum throughput recommended by manufacturers. The data will help inform country purchases and direct optimal deployment strategies of this technology.

## Research method and design

### PEPFAR procurement

Procurement data were collected from the PFSCM ORION procurement system. The ORION system is PFSCM’s integrated procurement system that controls, monitors and records all procurement activity for SCMS/PEPFAR. All figures represent the value of delivered commodities in US dollars ($) from September 2005 to June 2012. Data are categorised into pharmaceuticals (pharma; 66%), rapid test kits (9%), analysers and/or reagents (13%), laboratory consumables (lab; 8%) and ‘other’ (4%).

### CD4 cost-per-test analysis

We established a unit cost-per-test for the Benton Dickinson (BD) FACSCount™ and BD FACSCalibur™ instruments based on current prices paid by PFSCM and manufacturer-established consumption amounts.^7,8^ Consumption ratios were calculated for each product defined by the instrument manufacturer and end-user experiences (commodity list, consumption ratios and pricing in Table 1). Third-party control samples were not included in the overall pricing because of inconsistency in use. Total costs were then calculated based on the product requirements needed to conduct CD4 testing over a one-day period. Per-test costs were calculated using the following formula:

Cost-per-test = [(Σ (unit quantity commodity cost/usage rate per test)^A-E^ * tests per day) + daily control cost^*F*^]/tests per day {Eqn 1}

**TABLE 1:** CD4 count platform reagents and usage rates.

Item	Becton Dickson (BD) FACSCount™	Unit	Unit quantity	Usage rate and/or test
A	BD FACSCount Reagent Kit – CD4 (Single/Double tube, % – average price)	Test	50	1
B	BD FACSFlow Sheath Fluid (342003)	mL	2000	0.0825
C	BD FACSClean Solution (340345)	mL	5000	0.00125
D	BD FACSRinse Solution (340346)	mL	5000	0.00125
E	Thermal Paper Roll	roll	5	0.007
F	BD FACSCount Control Kit (340166)	Test	25	1 / testing day
**Item**	**Becton Dickson (BD) FACSCalibur™**	**Unit**	**Unit quantity**	**Usage rate and/or test**
A	BD Tri-Test CD3/CD4/CD45 with Tru Count Tubes	Test	50	1
B	BD FACS Lysing Solution	mL	100	0.2
C	BD FACSFlow Sheath Fluid (342003)	mL	100	0.0825
D	BD FACSClean Solution (340345)	mL	5000	0.00125
E	BD FACSRinse Solution (340346)	mL	5000	0.00125
F	BD Calibrite 3 Beads (340486)	Test	25	1 / testing day

Estimated costs do not include product wastage, start-up and shutdown product consumption, or any additional equipment maintenance costs, which can vary considerably by end users and across countries.

### Country-level cost analysis

Demographic, morbidity and service-statistics data were collected to inform multi-year (three- to five-year) laboratory instrumentation commodity requirements for three national quantification workshops in 2011 and 2012. Instrument test numbers were gathered through data collection exercises at all laboratory sites in countries A and B and at a subset of sites in country C. Data were provided by national laboratory leadership in each country, as well as PEPFAR implementing partners and United States government missions (USAID/US Centers for Disease Control and Prevention). Where information on instrument service interruptions as a result of commodity stock-outs was available, diagnostic consumption was adjusted in order to account for a reduction in the number of days of operation. Demographic and morbidity data, adjustments to test numbers associated with programme scale-up and general assumptions were documented. Final forecast estimates were then validated through consensus at each quantification workshop. Quantification outputs were used to develop first-year supply plans; to determine overall laboratory network commodity resource requirements, HIV diagnostics, care and treatment monitoring tests; and to prioritise laboratory spending when funding gaps were identified.

To determine the overall efficiency of CD4 testing instruments within the laboratory system, CD4 FACSCount™ and FACSCalibur™ testing data were extracted from the quantification and forecast data gathered during the country’s first-year forecast period. For each country, the number of individual instruments and where they were located within each national laboratory network were determined. We compared the number of tests by instrument with the manufacturer-recommended average instrument throughput capacity (50 sample tests per day for FACSCount™ and 350 per day for FACSCalibur™) to determine instrument utilisation rates.^7,8^ The rates were not adjusted for instrument breakdowns because of a consistent lack of data in each represented country at the time of data review. Diagnostic contribution was calculated by comparing individual machine throughput with total service provision estimates (service statistics), disaggregated by instrument type and level within the laboratory networks.

For Country C, we calculated product use to diagnostic contribution by the seven implementing partners using BD instruments (five partners using other brands were removed from the analysis). The total CD4 commodity cost was established for 2011 based on actual testing services provided during that year. The 2012 unit prices were calculated by projecting programmatic growth, planned instrument procurements and testing demand increases based on PEPFAR care and treatment targets.

## Results

### PEPFAR procurement

**FIGURE 1:**
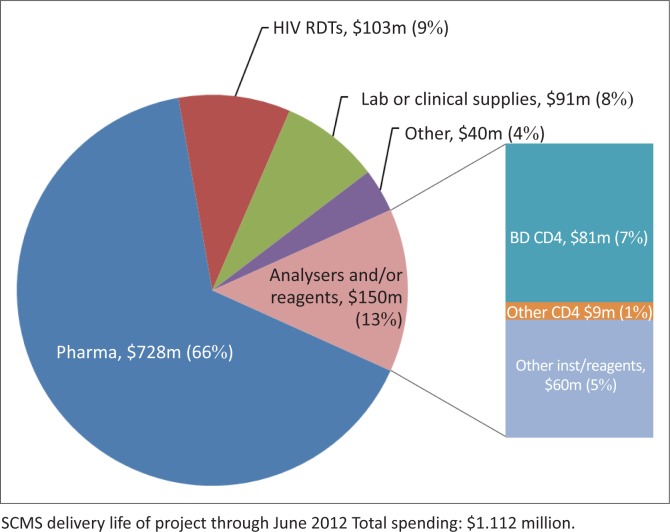
Partnership for Supply Chain Management (PFSCM) total spending as of June 30, 2012. PFSCM historical procurement data were used to determine the total expenditures for the Supply Chain Management Systems Life of Project (LOP). All figures represent the value of delivered commodities in US dollars from September 2005 to June 2012. Data are categorised into pharmaceuticals (Pharma [66%], HIV RDTs [9%], Analysers and/or reagents [13%], Lab/clinical supplies [8%] and Other [4%]).

**FIGURE 2:**
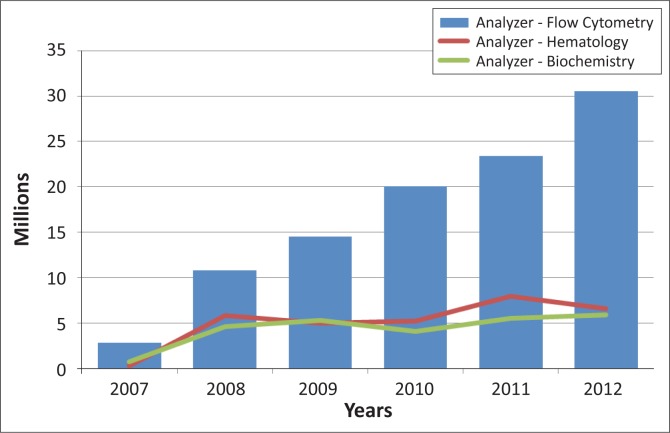
Partnership for Supply Chain Management (PFSCM) spending on laboratory commodities delivered through June 2012. PFSCM historical procurement data are displayed as expenditures per year from 2007 through June 2012. PEPFAR flow-cytometry expenditures show continual increases on a per-year basis.

As of June 30, 2012, PFSCM assisted in procuring pharmaceuticals (antiretrovirals, treatment for opportunistic infections) and other products with a value of about $1.1 billion (Figure 1). Thirty-four per cent of PFSCM’s overall commodity procurement, $384m, was spent on non-pharmaceutical products to supply PEPFAR-supported laboratories and testing sites in 53 countries. $150m of the non-pharmaceutical commodity amount was spent on reagents for analytical testing, with the majority ($90m) going to CD4 testing. Procurement of analytical testing products, specifically for CD4 testing, has increased by almost 20% annually for the past five years and in 2011 accounted for 8% of all procurement (Figure 2).

PEPFAR’s largest procurement of laboratory reagents during the period of September 2005 to June 2012 was for flow cytometry (64% of total reagent costs). BD products ($81m) represented 54% of the overall reagent costs and 90% of flow cytometry costs. For this reason, the two most commonly used BD flow cytometry platforms, FACSCount™ and FACSCalibur™, were chosen for further analysis in order to identify areas of cost savings or cost efficiency.

### Calculated CD4 cost-per-test analysis

We hypothesised that the cost for a test performed using these platforms would depend on volume, as was seen in a previous analysis.^9^ The CD4 cost-per-test analysis was limited to PFSCM prices paid for the reagents required for the BD FACSCount™ and FACSCalibur™ platforms (Table 1).

**FIGURE 3:**
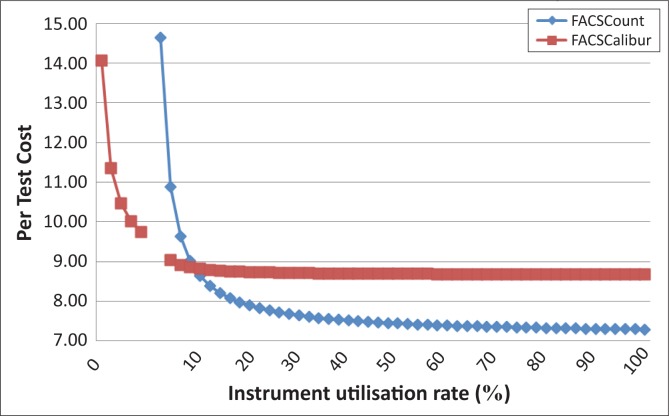
FACSCount™ versus FACCalibur™ utilisation price per test in US dollars. The reagent unit cost-per-test for the BD FACSCount™ and BD FACSCalibur™ instruments was based on historical Partnership for Supply Chain Management (PFSCM) pricing and manufacturer-established consumption amounts,^7,8^ using the reagents and consumption ratios in Table 1. Total costs were then calculated based on the product requirements needed to conduct CD4 testing over a one-day period. The rate of return diminishes to less than $0.01 per additional test added per day after a critical volume is achieved: FACSCount™, *n* > 20 tests or 40% throughput; FACSCalibur™, *n* > 45 tests or 15% throughput.

Using PFSCM pricing for necessary testing commodities, we found that a higher throughput did result in a lower cost-per-test, with the rate of cost savings decreasing as the volume approached the manufacturer-recommended maximum throughput of the instrument (Figure 3). The costs per test in this analysis were found to range from $14.64 to $7.29 for the FACSCount™ systems and $14.06 to $8.67 for the FACSCalibur™ system. We chose a rate of return of less than $0.01 per additional test per day as the point at which further investment in scale-up does not gain significant returns. Whilst in both cases the rate of return diminishes to less than $0.01 per additional test added per day after a critical volume is achieved, for FACSCount™ the volume must exceed 40% (*n* > 20 tests) of the maximum daily throughput (50 tests), whereas for FACSCalibur™, the critical volume is approximately 15% (*n* > 45 tests) of maximum throughput (350 tests).

Several variable costs drive the cost-per-test of the BD systems, especially at low throughput volumes. In this analysis, the BD control and/or calibration kit is an important cost driver for both instruments in that unit pricing reductions are based on maximising use of the control kit by increasing the volume of tests processed per day. In comparison with the FACSCount™, the FACSCalibur™ has an initially lower baseline cost-per-test CD4 reagent (Table 2). In fact, although the cost-per-test for both machines is similar at very low throughput, because of the lower baseline cost-per-test for FACSCalibur™ CD4 reagents, at extremely low volumes (*n* < 2) the FACSCalibur™ is slightly less expensive than the FACSCount™. However, as shown in Figure 3, for volumes of more than two tests per day, the cost-per-test of the FACSCount™ system drops below that of the FACSCalibur™ system. The FACSCalibur™ system maintains a unit pricing per test at about $1.40 higher than that of the FACSCount™ as efficiency is gained. These results indicate that for all BD platforms, cost savings can be achieved by maximising daily testing volumes per machine with optimal targets of 40% throughput for FACSCount™ and 15% throughput for FACSCalibur™ systems. Additionally and significantly, for volumes of tests of *n* > 2, the FACSCount™ system is the more cost efficient testing platform.

**TABLE 2:** FACSCount™ and FACSCalibur™ reagent unit price per test.

Samples	Days										
	1	5	10	15	20	25	30	35	40	45	50
FACSCount™ cost ($)†	14.64	8.64	7.89	7.64	7.51	7.44	7.39	7.35	7.33	7.31	7.29
FACSCount™ utilisation (%)	2	10	20	30	40	50	60	70	80	90	100
FACSCalibur™ cost ($)†	14.06	9.74	9.20	9.02	8.93	8.87	8.84	8.81	8.79	8.78	8.76
FACSCalibur™ utilisation (%)	0	1	3	4	6	7	9	10	11	13	14

† Amounts presented in US dollars.

### Country-level calculated cost analysis

#### Countries A and B

To estimate potential cost savings that might be achieved by maximising throughput volumes, data from two countries were used to compare existing and recommended targeted throughput in each country. The cost-per-test as a function of throughput, as described above, was compared with actual utilisation rates collected from testing facilities in two PEPFAR countries in order to calculate the average cost-per-test of a CD4 test performed on FACSCount™ machines. For FACSCount™, Country A (Table 3) is shown to maintain an aggregated average instrument utilisation rate of 47%, which translates to an average cost-per-test of $7.46. Country B (Table 4) is shown to have an average FACSCount™ instrument utilisation rate of 10%, resulting in a calculated average cost of $8.64 per test. Between Countries A and B, the average cost-per-test difference for tests performed using a FACSCount™ system amounts to an estimated $1.18 in unit pricing overall.

**TABLE 3:** Country A CD4 Testing Equipment Utilisation.

Country A	Machine numbers	Distribution (%)	Laboratory level	Utilisation (%)	Diagnostic contribution (%)	Estimated cost per test ($)¶
**All Instruments**	**138**		**All Levels**	**33†**	**100**	
FACSCount™	51	37	Primary outpatient	55†	40	7.41
FACSCount™	55	40	District Labs	44†	35	7.48
FACSCount™	12	9	Regional/Provincial Labs	22†	4	7.82
FACSCount™	5	4	National Reference Lab	53†	4	7.42
**FACSCount™ Total**	**123**	**89**	**-**	**47**	**82**	**7.46**
FACSCalibur™	0	0	Primary outpatient	0	0	
FACSCalibur™	3	2	District Labs	7	2	8.86
FACSCalibur™	7	5	Regional/Provincial Labs	8	5	8.84
FACSCalibur™	5	4	National Reference Lab	26	11	8.72
**FACSCalibur™ Total**	**15**	**11**	**-**	**14**	**18**	**8.77**

For FACSCount™ (cells marked with † are used effectively, ‡ are falling below ideal utilisation for Regional and/or Provincial Labs), Country A maintains an aggregated average instrument utilisation rate of 47%, a rate that maximises return on investment for the testing programme with an average per-test cost of $7.46.

¶ Amounts presented in US dollars.

**TABLE 4:** Country B CD4 Testing Equipment Utilisation.

Country B	Machine numbers	Distribution (%)	Laboratory level	Utilisation (%)	Diagnostic contribution (%)	Estimated cost per test ($)¶
**All Instruments**	**105**	**-**	**All Levels**	**11†**	**100**	**-**
FACSCount™	0	0	Primary outpatient	0†	0	-
FACSCount™	11	10	District Labs	7†	7	9.33
FACSCount™	51	49	Regional and/or Provincial Labs	9†	30	8.83
FACSCount™	2	2	National Reference Lab	33†	1	7.60
**FACSCount™ Total**	**64**	**61**	**-**	**10**	**38**	**8.64**
FACSCalibur™	0	0	Primary outpatient	0	0	-
FACSCalibur™	0	0	District Labs	0	0	-
FACSCalibur™	2	2	Regional and/or Provincial Labs	5	7	8.94
FACSCalibur™	7	7	National Reference Lab	15	25	8.76
**FACSCalibur™ Total**	**9**	**9**	**-**	**14**	**32**	**8.77**

For FACSCount™ (cells marked with † require attention, below ideal utilisation), Country B maintains a low aggregated average instrument utilisation rate of 10%, with an average per-test cost of $8.64.

¶ Amounts presented in US dollars.

To identify areas in which the greatest cost efficiencies could be gained through increased utilisation of CD4 testing equipment within the tiered laboratory system, we performed a more targeted analysis of Country B. In our review of the data for Country B, we observed that laboratories in regional and provincial laboratories had 51 FACSCount™ machines. These sites had an utilisation rate of 9% (*n* < 5 tests per day), but contributed 30% of the total CD4 tests conducted for the country (Table 4). Costs in these laboratories averaged $8.83 per unit test. According to our analysis of cost-per-test as a function of instrument utilisation, Country B would achieve a cost-per-test of $7.89 were it to increase its utilisation of these machines to 20%; a per-test savings of $0.94. Moreover, were Country B to increase utilisation to 40%, the target for maximising efficiency, the cost-per-test would be $7.51, a per-test savings of $1.32. Extrapolating the savings accrued by Country B for increasing CD4 equipment utilisation to 20% or 40% in regional and/or provincial laboratories as a percentage of the total budget spent on CD4 testing would reduce expenditures by 14% and 17%, respectively. Overall, small increases in utilisation rates, when targeted to facilities with large diagnostic contribution to the total number of tests performed, can result in significant cost savings.

#### Country C

In country C we calculated CD4 test costs by seven different implementing partners. Targeting implementing partners with high CD4 testing contribution and seeking ways to increase utilisation rates may be one way to reduce testing costs and establish further commodity consumption efficiencies. The analysis of implementing partners in Country C provided an opportunity to investigate implementing partner CD4 testing contributions for PEPFAR in 2011 and to examine projected growth into 2012. Partners 2, 4 and 5 use FACSCount™ machines and contribute significantly to CD4 testing services within the PEPFAR Country C programme for 2011 and 2012 (Table 5). Instrument utilisation rates for Partners 2, 4 and 5 are, respectively, 30%, 4% and 43% in 2011 and 5%, 4% and 57% in 2012.

**TABLE 5:** Country C Comparison of CD4 Testing Equipment Utilisation in 2011 and 2012. For Country C, product use and diagnostic contribution were disaggregated according to those implementing partners using BD instruments (five partners using other brands were removed from the analysis). Total CD4 commodity costs for 2011 were based on actual testing services provided. 2012 unit prices were based on projected programmatic growth and planned instrument procurements. Instrument utilisation rates for Partners 2, 4 and 5 are, respectively, 30%, 4% and 43% in 2011 and 5%, 4% and 57% in 2012, indicating appropriate growth for Partner 5, but a decrease or no increase in efficiency (reduced cost-per-test) for Partners 2 and 4.

Partner	2011				2012					
	FACSCount™ tests	FACSCount™ utilisation (%)	Cost and/or test ($)^¶^	Diagnostic contribution	FACSCount™ tests	FACSCount™ utilisation (%)	Cost and/or test ($)^¶^	Diagnostic contribution	Testing increase (%)	Spending increase (%)
2	41 332	30	7.64	11.9	55 798	5	10.27	12.5	35	45
3	7800	34	7.58	1.5	15 451	68	7.36	2.3	98	48
4	82 527	4	10.89	12.8	95 116	4	10.89	11.1	15	13
5	156 825	43	7.49	16.9	258 427	57	7.40	20.9	65	27
7	18 232	10	8.64	2.0	18 232	10	8.64	1.5	0	0
10	530	2	14.64	0.0	2470	11	8.52	0.6	366	63
12	28 140	5	10.27	3.0	37 055	6	9.96	3.0	0	22
**Total**	335 386	-	-	-	482 549	-	-	-	44	25

¶ Amounts presented in US dollars.

BD, Becton Dickinson.

The model for optimal instrument utilisation is Partner 5, with a cost-per-test average of $7.49, which further gained efficiency in 2012 by increasing instrument utilisation to 57% with an average per-test cost of $7.40.

In contrast, Partner 4 had a throughput of 4% capacity in 2011 and 2012, with a calculated cost of $10.89 per test. Table 5 indicates that the projected number of tests performed has increased substantially (by 13%), yet the percent utilisation remains unchanged. This indicates that, rather than increasing throughput on existing machines, additional machines have been procured by this partner such that an exceedingly high cost-per-test is maintained over time (10 new FACSCount™ instruments planned for 2012). Given the extremely low throughput observed for Partner 4, even a 2% increase in utilisation (an average of one more test per day) could reduce per-test costs by over $1 ($10.89 − $9.64). Here, consolidating testing into existing machines to increase efficiency rather than procuring new equipment would result in significant cost savings over time.

Similarly, in 2011 Partner 2 operated with an average instrument utilisation of 30% and a cost-per-test of $7.64 (Table 5). Ideally, for 2012, Partner 2 would increase instrument utilisation to 40% to gain efficiency and lower the cost-per-test for its CD4 testing programme. However, in this case, despite a significant projected increase in tests performed, instrument utilisation decreases to 5%, giving an average cost-per-test in 2012 of $10.27. This is an increase of $2.63 per test over 2011 costs. Once again, the decrease in utilisation likely indicates the procurement of additional equipment (43 new FACSCount™ instruments planned for 2012) within a small testing pool that reduces the average volumes for all machines operated by Partner 2. Using the 2011 per-test cost ($7.64) to calculate the budget needed for Partner 2 to perform the 55 798 tests projected for 2012 results in an estimated budget of $426 297 for reagent procurement. In this case, the decrease in utilisation and subsequent increase in per-test cost in 2012 (to $10.27) results in an actual budgetary requirement of $573 045. The underutilisation of CD4 testing equipment results in an additional $146 748 in reagent procurement needed to perform the same number of tests. These results indicate that efficient systems seeking to expand coverage must consider the cost implications of reducing volumes through existing equipment.

## Discussion

Our work in reviewing procurement and participation in laboratory commodity quantification exercises indicates that cost savings can be realised with better utilisation of CD4 testing instruments. In this analysis of national quantification exercises in three countries, we identify one area in which greater efficiency may be established by maximising CD4 instrument utilisation rates to reduce the cost-per-test of CD4 testing (represented by BD FACSCount™ and FACSCalibur™). These results indicate that for all BD platforms, cost savings can be achieved by maximising daily testing volumes per machine with optimal targets of 40% throughput for FACSCount™ and 15% throughput for FACSCalibur™ systems. This is particularly important for testing sites with large diagnostic contribution where small increases in utilisation rates can result in significant cost savings. For programmes seeking to expand coverage, acquisition of additional CD4 machines should consider the need to increase utilisation by consolidating testing into existing machines where existing referral networks are adequate.

Considering instrument placement before procurement, including accurate estimation of the appropriate demand at deployment locations, ultimately increases consumption efficiencies and reduces overall costs. To that end, it is particularly important to understand the cost implications of decentralising services to sites that underutilise instruments that contribute little to overall diagnostics. Underutilising instruments that contribute less to the overall testing uptake has less of an impact on overall commodity costs, whereas underutilising instruments that have higher overall testing contributions has a higher impact on cost. For example, a FACSCount™ instrument operating at $9.00 per test that contributes to only 4% of the national testing target will have a lower impact on overall programme costs than if that same machine were contributing to 35% of the national testing targets. Strategically relocating existing instruments could improve utilisation, as could replacing underutilised equipment with lower capacity point-of-care (POC) tests. It is important to either match site-level demand to the instrument (placing smaller instruments in low-volume clinics) or to place underutilised instruments into higher volume sites as back-up instruments in order to add redundancy in the event of equipment breakdown. Planned expansion should first seek to efficiently utilise existing equipment and minimise the appropriation of tests from currently functioning sites.

Throughput of a selected CD4 testing machine should match testing consumption at the service delivery point. For certain facilities, diagnostics consumption falls below the optimal throughput volume for the more commonly-used CD4 platforms into the range of the low-throughput POC CD4 testing platforms, for which the cost-per-test is a flat rate from a commodity consumption perspective and does not vary with volume. In these cases, it may be more cost efficient to use a POC CD4 testing platform. This decision would require selecting a single supported POC instrument that is included in the national health laboratory strategic plan and strategically integrated into the tiered laboratory network to optimise existing instrumentation, balancing access to service.

It should be noted that this analysis shows that for nearly all levels of throughput, the FACSCount™ platform is less expensive on a per-test basis than the FACSCalibur™ system. Choosing to implement the FACSCalibur™, however, does have some benefit in very high-throughput facilities because it is fully automated and processes samples more quickly than the FACSCount™. Thus, use of the FACSCalibur™ has the potential to reduce overall laboratory costs by allowing for higher testing volumes at service provision sites with less dependence upon laboratory staff time.

Whilst an acceptable level of instrument utilisation may appear to be achieved at the national level, for some countries disaggregating instrument utilisation for CD4 testing equipment by tiered level, region, or possibly by implementing partner, can assist in developing a more targeted intervention. For example, at the level of an individual implementing partner, diagnostic throughput may be very low, resulting in a high cost-per-test for testing sites managed by that implementing partner. For these partners, it may be useful to consolidate testing to existing equipment or to consider the suitability of new equipment for proposed expansion sites. Further comparative analysis could potentially guide appropriate instrument deployment based on the site-specific demands, maximising partner cost efficiencies and further reducing the overall CD4 testing unit pricing scheme.

## Limitations of the study

The study presented here has several limitations. The first is that the cost-per-test analysis considers only the cost of reagents. This likely results in an underestimation of the true cost-per-test, which would also include the cost of the equipment, human resources, service maintenance contracts and of expired and wasted product because of low equipment throughput and instrument breakdown. The second limitation to the analysis is a lack of information on efficiencies gained at very-low-volume sites by batching test samples collected over several days. Very-low-volume sites using batch testing would at some level increase throughput and decrease the daily cost-per-test, perhaps significantly, at very low volume. Finally, the analysis centres on BD CD4 testing platforms; whilst these are the most commonly-used CD4 testing platforms in PEPFAR-supported HIV testing programmes, other platforms with considerably lower reagent costs are used, albeit far less commonly, throughout Africa.

### Recommendations

The 2008 Maputo Declaration on Strengthening of Laboratory Systems^6^ called for harmonisation and standardisation of tests, reagents, consumables and instruments at each level within a tiered laboratory system. Since that time, harmonisation has proven to be a challenge for many reasons, including evolving diagnostic coverage during scale-up, system maturation, existing procurement policies and changes in demographic and morbidity demands. Nonetheless, in support of the Maputo Declaration, we must pursue a strategy of optimising laboratory procurement through informed decision making in order to advance harmonisation and maximise consumption efficiencies. Such a strategy can advance harmonisation at all levels within the tiered system by using site-level data that informs the selection of equipment based on need or consumption at the point of use (consumption efficiencies); including the platform within the National Testing Algorithm, if one exists (HIV, tuberculosis and malaria); and understanding the sustainability of the platform within the regional setting (adequate infrastructure, reagent supply, maintenance and training curricula). The overall objective of optimising laboratory procurement is to develop instrument placement strategies that will increase appropriate coverage, increase overall commodity consumption efficiencies and ensure that instruments are operational, accounted for and, ultimately, maximise return on investment.

As donor support and financial resources must stretch further to meet mounting global health needs, it is critical that potential savings be sought wherever possible. Recognising the commitment and financial obligation required to support instrument life cycles amongst many competing priorities requires a broad perspective and regular re-evaluation of needs. In this way, translating historical laboratory procurement and quantification data into service performance indicators across all laboratory instrumentation and across different platforms can provide visibility into the critical operational aspects and create opportunities to establish further efficiencies of a laboratory network over time. Product consumption and testing numbers can identify commodity wastage, supply chain management inefficiencies and the underutilisation of machines based on their potential testing capacity. Armed with this information, those directing and managing laboratory networks are better equipped to develop responsive laboratory optimisation strategies that enable the best use of every donor dollar.

## Conclusion

Donors, implementing partners and host-country governments must make deliberate, transparent and coordinated efforts to advance evidence-based laboratory optimisation processes. Countries must be positioned to take into account how best to balance costs, increase consumption efficiencies and ensure overall access to services. To realise further cost savings and continue to increase access to patient testing services, the development of laboratory optimisation strategies should serve as a critical step in further advancing overall healthcare delivery. Fundamental to this strategy is ensuring a clear understanding of how effectively laboratory-related commodities are consumed in support of healthcare programmes.
